# The Effect of Work Safety on Organizational Social Sustainability Improvement in the Healthcare Sector: The Case of a Public Sector Hospital in Pakistan

**DOI:** 10.3390/ijerph18126672

**Published:** 2021-06-21

**Authors:** Zia Ullah, Mohammed Ali Bait Ali Sulaiman, Syed Babar Ali, Naveed Ahmad, Miklas Scholz, Heesup Han

**Affiliations:** 1 Leads Business School, Lahore Leads University, Lahore 54000, Pakistan; 2 Department of Marketing and Entrepreneurship, Dhofar University, Salalah 211, Oman; msulaiman@du.edu.om; 3 Department of Accounting & Finance, Faculty of Management Sciences Salim Habib University NC-24, Deh Dih, Korangi Creek, Karachi 74900, Pakistan; babar.ali@shu.edu.pk; 4 Faculty of Management Studies, University of Central Punjab, Lahore 54000, Pakistan; naveeddgk2010@gmail.com; 5 Department of Building and Environmental Technology, Division of Water Resources Engineering, Faculty of Engineering, Lund University, P.O. Box 118, 221 00 Lund, Sweden; 6 Department of Civil Engineering Science, School of Civil Engineering and the Built Environment, University of Johannesburg, Kingsway Campus, P.O. Box 524, Aukland Park, Johannesburg 2006, South Africa; 7 Department of Town Planning, Engineering Networks and Systems, South Ural State University (National Research University), 76, Lenin Prospekt, 454080 Chelyabinsk, Russia; 8 College of Hospitality and Tourism Management, Sejong University, 98 Gunja-Dong, Gwanjin-Gu, Seoul 143-747, Korea; heesup.han@gmail.com

**Keywords:** social sustainability, public hospitals, work safety, safety training, safety policies

## Abstract

Social sustainability is the much emphasized organizational phenomenon in Western literature; however, in emerging economies, its importance has only been realized in the recent past. Social sustainability is the amiability of the relationship between employees and the organizations on a relatively permanent basis. Social sustainability is the key determinant of organizational sustainability and organizational effectiveness. As healthcare organizations are labor-intensive, the role of social sustainability in hospitals is more crucial. The purpose of the present study is to understand the role of work safety in improving social sustainability in public sector hospitals. To this effect, we collected data from 431 healthcare professionals of a large public sector tertiary and teaching hospital in the city of Lahore Pakistan and analyzed the data using structural equation modeling (SEM). The results uncovered certain important facts, which were not expected per se. Job design, coworkers’ behavior towards work safety, and supervisors’ role in ensuring work safety are the key factors that influence social sustainability. However, surprisingly, in the eyes of employees, management practices and safety programs/policies do not contribute to the work safety of the hospital under study. Keeping in view the findings, we suggest that management must participate in work safety affairs directly and formulate indigenous policies and programs according to local needs. Job analysis is needed to redesign job structures to meet workplace safety requirements. Formal and informal training will be beneficial to make workers and supervisors more aware, more sensitive, and more responsible regarding work safety.

## 1. Introduction

The concept of organizational is explained with the triple bottom line (TBL), which categorizes organizational sustainability into economic, environmental, and social groups [1]. The social sustainability of an organization is related to the cordiality and amiability of the relationship between organizations and their stakeholders, particularly the employees. Organizations are socially sustainable with the productive relationship with employees that is relatively permanent and durable in nature. The resource that is highly scare and not easily available in the market is a competent and motivated worker. That is why contemporary organizations pursue sustainable competitive advantages through sustainable human resources. Hence, social sustainability is achieved through human development, including education, training, conducive work environment, appropriate compensation, and sound corporate culture.

The healthcare sector is one of the most important institutions of a state, directly involved in public welfare. A nation as a whole needs healthcare services irrespective of its socio-economic standings [2]. As healthcare is a labor-intensive industry, the quality and flow of its services are contingent upon the quality of its employees’ performance [3]. There is no differing opinion that healthcare services are very sensitive and error of any kind cannot be afforded as such as it could cost a human life [4,5]. In a nutshell, the entire quality of healthcare services is the reflection of the competence and commitment of its workforce. The healthcare system aims to promote, maintain, restore, and improve the health indicators of a nation [6]. However, healthcare organizations always remain under pressure for multiple reasons [7]. Demand for healthcare services is continuously on an increasing trend owing to the asymmetric increase in population and healthcare facilities, particularly in developing economies [8]. In resource-deficient or resource-mismanaged countries, the prevalence of morbidity and diseases is relatively high and healthcare provisions are not all perfect [9]. Hospitals are generally observed to be overloaded and serving many patients beyond their capacities [10].

Under such circumstances and specifically within the given context, the seemingly effective intervention is developing and improving the social sustainability of hospitals and other healthcare organizations [11]. Researchers, administrators, practitioners, and policymakers have mainly focused on other aspects of sustainability including economic and environmental aspects, and giving the least attention to the social aspect of sustainability [12,13]. It is crucial and the need of the day to explore socially sustainable healthcare models to achieve effectiveness and efficiency in the service [14,15,16]. Healthcare organizations have multiple stakeholders including government agencies, suppliers, patients, scientists, pharmaceutical companies, and healthcare professionals [17]. Healthcare professionals (clinical and non-clinical) are the frontline actors and they influence the overall service provision quite profoundly [7].

The prevalence of work-related safety issues is common in the world, but it is more serious in developing countries in particular [18,19]. Many employees sustain physical or mental damages in performing their jobs and they carry the consequences out to their families and immediate social circle [20]. The consequences could be loss of dignity, depression, anxiety, premature aging, attempt at suicide, low self-esteem, lack of trust in people, absenteeism, losing autonomy, injuries, and physical and musculoskeletal injuries [21]. Healthcare work settings are more complex and healthcare workers, apart from other job-related issues that are common across organizations, are prone to health-related issues [18]. Hospitals’ environments contain health hazards including ergonomic, chemical, biological, and physical hazards that add to work-related difficulties [22]. Employees, as such, expect job safety, coworker safety, supervisor safety, management safety practices, and safety programs to be in place in their work environment [23]. Employees’ belief in the effectiveness of the safety system has direct bearings on their performance and ultimately determines the strength of their relationship with the organization [11].

The professionals working in healthcare organizations, specifically in hospitals, are relatively more prone to health- and job-related issues [24]. Employees with more job-related issues are likely to experience job dissatisfaction and turnover intentions [25]. These two attitudes simply destroy employees’ pro-organizational behaviors that an organization wants to reinforce. Organizations direly need to promote positive emotions of employees and the recurrence of positive emotions usually shapes social sustainability.

Keeping in view the relatively excessive work safety issues in healthcare settings and the dominant and decisive role of human resources in the provision of healthcare services, this study is designed to investigate such a sensitive issue where lifesaving services may be compromised owing to possible disturbance in social sustainability. Thus, this is an explanatory kind of study that aims to testify to our assumption that work safety is one of the dominant factors in developing and improving social sustainability in healthcare organizations. The reason it felt necessary to carry out this study is that many studies found precarious work safety issues in healthcare settings within similar contexts. Precisely speaking, this study aimed at understanding the fluctuation in the nature of the relationship between employees and the organization due to safety conditions prevailing in the workplace. Workplace safety was classified into five dimensions, including job safety, coworker safety, supervisor safety, management safety practices, and safety programs. Thus, we studied the links of these five dimensions with organizational social sustainability. Our hypotheses present that each of the five dimensions had a role in the formation of social sustainability. We chose hospitals for data collection because of the relatively high prevalence of work safety issues in hospitals. Data were collected from four hundred and thirty healthcare professionals working in different hospitals in Lahore city of Pakistan and structural equation modeling was applied to test the hypotheses. All the hypotheses were accepted, except the relation of management safety practices with organizational social sustainability (OSS). Work safety issues do exist in the workplace and can be managed through job restructuring, training of coworkers and supervisors, apparent involvement of senior management, and effective formulating and evaluating of safety programs and policies. The study was limited to the collection of data from a single teaching hospital and cross sectional in nature. It is suggested that future research should expand data collection to many hospitals including the private and public sector, as well as teaching and non-teaching hospitals, to enhance the generalizability of the findings.

## 2. Literature Review

Sustainability is a buzzword that is very flexible and, apparently, everything can be associated and hyphenated with it [26]. This is why sustainability lacks a sustainable definition. The two prime challenges of sustainability are the lack of an agreed-upon definition of the term and the multiplicity of interchangeable words used by researchers [27]. Frequently used alternate terms instead of sustainability include continuation, maintenance, routinization, durability, and institutionalization [28,29]. The definitional variations are the result of multiple disciplines (for example, sociology, psychology, health systems, management sciences, prevention science, justice, and education) [30] confronting similar problems. For the definition of the construct with particular reference to healthcare, sustainability was crafted to encompass these five key constructs: (1) after a specified period of time; (2) the programs, clinical interventions, and/or implementation plans continue to be delivered and/or (3) people’s behavior change (i.e., patient, clinician) is maintained; (4) the program and individual behavior change may evolve or adapt while (5) continuing to produce benefits for individuals/systems [31].

Sustainability in organizations has been an attractive subject in academic circles, corporate meetings, political platforms, and research forums [32,33]. Discussions and deliberations on what sustainability is, why is it imperative, and how to obtain it are prevalent [34]. Evaluations of organizational success have gone beyond effectiveness and efficiency, which were aimed at profitability, and in present times, sustainable organizations are considered as successful organizations. Sustainability is a composite construct and it is the integration of environmental, social, and economic components into the organization [35,36]. A sustainable organization does not necessarily succeed in becoming financially strong, but rather in effectively balancing prosperity, people, and the planet by guaranteeing a vibrant equilibrium among these 3 P’s [37]. Sustainability in an organizational context represents a perpetual process rather than a state of perfection. It is like greenery, it nurtures and flourishes when watered and cared for, but dies away quickly if it is not [38].

The sustainability literature normally approaches sustainability as either a meso-level organizational concept or a macro-level societal one. Even though precise and consensus-based definitions of sustainability are not available, most researchers mention three interrelated dimensions of sustainability i.e., economic, social, and environmental [39,40,41,42,43]. However, much attention is given to environmental and economic concerns, thus making them dominant dimensions [35,44,45], paying the least attention to the social aspect of sustainability. The norm of reciprocity forms social sustainability by enhancing trust and cooperation in a group of people and describes this complex relationship. To study macro-level sustainability, researchers are paying more attention to the social aspect of sustainability [46,47,48]. The social sustainability of an organization is the ability of employees to work under any situation faced individually and collectively. So, the important aspect of the sustainability of an organization is the integration between its employees [49]. An organization cannot become sustainable and grow complex if the employees develop individuality without proper integration [50]. Similarly, any integration attempt inside an organization without any development in individual employees does not make sustainability certain, but creates instead a weird way of collaboration among employees who have no novelty to share with each other [51]. Firstly, members of the organization should grow complex in their thoughts and actions and make their efforts worth the struggle and significant for organizational sustainability. Secondly, the employees shall have the ability to learn collectively and become integrated into groups, departments, and an organization with complex mental models and action patterns [52]. Thus, organizational social sustainability comes into existence with a linkage of interacting individual employees and groups whose development grows together [53].

The prevalence of workplace safety issues is a common phenomenon in the world and it is more serious in resource-poor regions in particular. Many employees are affected physically and mentally by working in an unsafe work environment and even carry the consequences to their families and immediate social circles. An occupational hazard is an injury or ailment resulting from the work one does or from the surrounding in which one works [54,55,56]. The consequences of workplace hazards could be trauma, even post-traumatic stress disorder (PTSD), loss of dignity, anxiety, depression, attempt at suicide, decreased self-esteem, lack of trust in people, premature aging, losing autonomy, injuries, absenteeism, and physical and musculoskeletal injuries [57,58].

Based on the analyzed literature, it is stated in this research that healthcare settings are relatively the least safe work environment as chemical, biological, and ergonomic factors frequently exist, exposing the workers to health issues. The literature on work safety in healthcare is mainly focused on those workplace safety issues that endanger the health of the worker. To the best of our search, we did not find literature on the role of work safety in the development of organizational social sustainability in the healthcare industry. Furthermore, there is rich literature on occupational hazards and workplace health hazards, and certain other dimensions of work safety like job safety, supervisor safety, coworker safety, management safety practices, and safety program implementation have not been collectively studied concerning social sustainability. From the literature review, questionnaire reference, and following the literature citied, this is not new work, but these types of survey and analysis are often useful at different locations and facilities to study the different mindsets of the employee and employer.

## 3. Theoretical Framework and Hypotheses

Work safety involves the relationship between employees and work; equipment, materials, and technology; and economic considerations like productivity and the environment. Preferably, work should be health-promoting, not harmful to physical and mental wellbeing, and not unreasonably difficult. For economic reasons, as high a level of productivity as possible must be achieved. The ABC theory states that the attitude, behavior, and conditions that follow as a result of encountering risk factors result in a change of behavior [59]. In fact, everyone is motivated differently, thus understanding safety motivation in individuals becomes critical for long-term change of behavior [59,60]. The theory states that the typical hazards are structural, sociological, biological, mechanical, electrical, chemical, and physical hazards [60,61,62,63]. People’s behavior in an organization is perhaps one of the major determinants of workplace safety, particularly as employees interact within a host of diverse safety issues [64]. Thus, human behavior plays a big role in a task accomplished by an employee. The task may have a positive and a negative impact on the connection to the employee doing the particular task. The norm of reciprocity [65] explains that people repay in kind what organizations do for them. The norm of reciprocity shapes social sustainability by increasing trust and cooperation in any group of people and explains this complex relationship. In addition to the empirical evidence supporting the norm of reciprocity, there are good theoretical reasons for why our behavior should be regulated by such a norm [66].

The composition of organizational sustainability consists of three dimensions—economic, environmental, and social [67,68]. This study considers the social aspect of organizational sustainability. Work safety is a multidimensional phenomenon that includes physical, psychological, economic, and social aspects. Most of the studies in the field of work safety have confined themselves to occupational health hazards including physical, chemical, and ergonomic hazards that have direct bearings on the health of employees. This study encompasses relatively broader aspects of work safety in order to have an exhaustive view of it.

### 3.1. Definition of Variables

#### 3.1.1. Organizational Social Sustainability (OSS)

Organizational social sustainability was chosen as the criterion variable for this study. Social sustainability is one of the three dimensions of sustainability [69]. The collective impact of employees on sustainability and their role in developing it is basically referred to as social sustainability [69,70]. It is a reciprocal function as and when an organization provides a conducive work environment by meeting the expectations of employees and employees in return meet organizational expectations on a long-term basis [71]. Thus, we define the term social organizational sustainability as the amiability of the relationship between the employees and the organization on a relatively permanent basis.

#### 3.1.2. Work Safety (WS)

This refers to the working environment, encompassing all factors that affect the health, safety, and wellbeing of workers [72,73]. It is a multidisciplinary field that deals with the health, safety, and welfare of people at work. The goal of work safety is to provide employees with a healthy work environment where they enjoy physical, psychological, economic, and social safety while performing their jobs [74]. We chose five factorially distinct variables, including job safety, coworker safety, supervisor safety, management safety practices, and safety programs, from the study of Hayes et. al. [75]. However, we define these variables in the following way:

i. *Job Safety*: Job safety indicates the integration of all the measures considered necessary to free the work environment of physical, psychological, and social ills. Job safety is ensured when it is not dangerous, hazardous, unsafe, unhealthy, scary, and risky, and there is no fear and chance of death.
ii. *Coworker Safety*: Coworker safety refers to the safety orientation of the workforce. It is the seriousness and concern of the workforce about the safety of other employees working in the same workplace. It is employees’ overall obedience to safety rules, caring about others’ safety, encouraging others to be safe, and making efforts to keep the work environment free of hazards.
iii. *Supervisor Safety*: This is the behavior of the immediate supervisor towards safety measures. It is the extent to which a supervisor is serious and concerned regarding making the work environment safe. Safety-conscious supervisors encourage employees’ safe behavior, implement safety rules, explain safety rules and processes, reward safety behaviors, and involve employees in planning and implementing safety measures.
iv. *Management Safety Practices:* This is about management intervention to ensure work safety and provision of a hazards-free work environment to its workers. It is assessed through the provision of safety training, safety inspections, provision of safety equipment, quick response to safety problems, rewarding safe workers, and provision of safety information.
v. *Safety Programs and Policies:* This explains the relevance and effectiveness of safety programs and policies. To ensure safety at work, policies should be clear, useful, worthwhile, applicable, valid, and reliable.

### 3.2. Operationalization of Variables

Since all the variables involved in this study are construct in nature, that needed to be transformed into quantifiable form. We assigned indicators (items measureable at five points likert type scale) to each variable to measure them (Table 1).

### 3.3. Hypotheses

**Figure 1:**
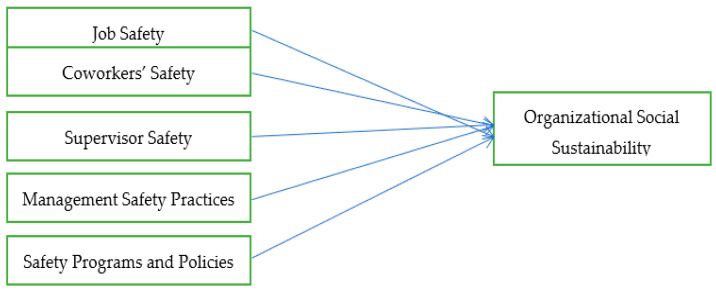
Schematic view of research model.

Contemporary organizations cannot afford to overlook and neglect organizational social sustainability. Globalization, technological advancement, and trade agreements have made access to financial and technological resources easily possible for all organizations across the globe [76,77,78]. So, financial and technological resources are no longer sources of competitive advantage [79]. As motivated, loyal, and competent employees are not readily available in the market as such, managers seek competitive advantage through their people [80]. There are many organizational and other factors that directly or indirectly impact employees’ attitudes towards their organizations [81]. For example, monetary and non-monetary rewards, career prospects, vertical and horizontal relationships, appropriate work conditions, and organizational reputation have direct effects on employees’ job satisfaction and turnover intentions [76]. Workplace safety is one of the key variables that profoundly influence employees’ decision to remain with the organization or otherwise [82]. In most cases, the effects of workplace safety issues remain even after retirement. Thus, employees rationally evaluate workplace safety conditions while making career-related decisions. Keeping in view these facts, we formulate the following hypotheses that have also been shown diagrammatically (Figure 1):

**Hypothesis** **1** **(H1).** *Job safety will positively influence the improvement in organizational social sustainability.*

**Hypothesis** **2** **(H2).** *Coworkers’ attitude toward workplace safety will directly influence the improvement in organizational social sustainability.*

**Hypothesis** **3** **(H3).** *Supervisors’ attitude and behavior towards workplace safety will directly influence the improvement in organizational social sustainability.*

**Hypothesis** **4** **(H4).** *Management safety practices to make the workplace safe will positively influence the improvement in organizational social sustainability.*

**Hypothesis** **5** **(H5).** *The soundness of organizational safety programs and policies will positively influence the improvement in organizational social sustainability.*

## 4. Methods

The proposed model is tested in a healthcare setting. A large public sector tertiary hospital (having 1299 beds) situated in the city of Lahore Pakistan was selected for data collection. The hospital has 4970 employees working in 42 different departments. The hospital provides healthcare services to around 1.60 million patients annually. In total, 20% of these patients are admitted to the hospital for further investigation and treatment. Eighty thousand surgeries are carried out and 2.2 million laboratory tests are conducted each year. As the hospital is state-owned and financed out of public money, ninety percent of healthcare services are provided free of cost. The hospital is attached to a medical university that offers graduate and postgraduate medical programs and the hospital and its patients are used for clinical training of the medical students.

Approvals for data collection were obtained from the vice-chancellor of the university and the chief operating officer of the hospital. A written declaration was submitted to the ethical committee of the hospital to follow ethical standards during data collection. In addition to this, informed consent was obtained from each respondent for participation in the survey voluntarily. The chief operating officer instructed the director of the research and development (R&D) department of the hospital to help the authors to gather data. The director R&D nominated seven employees of the hospital including two medical officers, three nurses, and two data and computer operators to collect data on our behalf. One of our authors provided necessary training on the administration of the questionnaire, on answering respondents’ expected queries, and data collection. The authors were asked to stay out of the hospital owing to the aggressive prevalence of the COVID-19 pandemic. A total of 568 questionnaires were administered to the randomly selected sample and 431 questionnaires, validly filled out, were included for statistical analysis. Hence, the response rate for this data collection remained close to 76%. The demographic characteristics of the respondents are depicted in Table 2.

### Measures

The authors adopted previously used and quality verified scales. To measure work safety (WS), the scale designed by Hayes et al. [75] was used. This instrument has five variables to measure work safety and it is known as the work safety scale (WSS) questionnaire. Thirty-one items were used to measure the five dimensions and a five-point Likert type scale was used to collect responses. The scale used to measure organizational social sustainability (OSS) was taken from Cella-De-Oliveira [83]. The instrument developed by the author measures social sustainability in organizations using six items. Although the instruments were already tested for validity and reliability, we further tested them to establish the quality of the data. Apart from reliability, convergent validity, and discriminant validity, we tested for autocorrelation, multicollinearity, and common method bias. We found the data to be free of these kinds of discrepancies.

The responses were analyzed from two angles. Firstly, statistical treatments were applied to check the quality of data and test the hypotheses. Secondly, the responses obtained against each item were analyzed. Much of the information in the discussion section and recommendations reflects the responses to the questions.

The demographic information of the respondents (Table 2) shows that our sample was quite representative. Both genders participated in study and employees from 18 to 60 years of ages participated in the study. Respondents were classified age-wise into five categories. The majority of our respondents were 26–40 years of age and respondents from all age groups participated in the study. As per experience, the sample was grouped into five categories. Most of the respondents had 6–20 years of job experience. According to profession, doctors and nurses almost equally participated in the study, while 13 percent were other employees.

## 5. Results

### 5.1. Reliability

Although we used already tested scales, we verified the reliability and validity of the instruments to avoid any discrepancies that may come into play owing to changes in the context and respondents. We measured reliability through Cronbach and composite reliability values (Table 3). Cronbach’s alpha values for all the variables are above the threshold value of 0.70, confirming inter-rater reliability and, in the same way, composite reliability values for all six variables are higher than the cutoff value, which is also 0.70. These two indicators are considered to be sufficient to measure the reliability of an instrument [84].

### 5.2. Validity

Validity determines how accurately an instrument measures what is supposed to be measured. We measured convergent validity using average variance extracted (AVE) values. The threshold value for AVE is 0.50 [85] and all the AVE values shown in Table 1 are above the cut-off value; so, convergent validity is established. We tested for discriminant validity using the Fornell–Larker criterion and Heterotrait–Monotrait (HTMT) ratios as indicated by Henseler, et al. [86]. As per the Fronell–Larcker criterion, indicator(s) should better explain its own latent variable rather than the variance of other latent variables [87]. Thus, each construct possesses a greater value than the correlations of other latent variables establishing high discriminant validity (Table 4). According to the HTMT criterion, a value above 0.90 shows the lack of discriminant validity [88], while in our study, all the HTMT values are below the cutoff point (Table 5). Additionally, variance inflation factor (VIF) statistics ruled out the possibility of common method bias (CMB) and multicollinearity in our data, as all the VIFs are below 3.30 (Table 6).

### 5.3. Correlations

The correlation matrix explains the direction and intensity of the association of variables. It also explains the existence of autocorrelation in the data. A correlation value at 0.70 is considered a strong association [89], while a value higher than 0.80 indicates the existence of autocorrelation [90]. All the correlation coefficients extracted from our data (Table 7) are above 0.60 and positive, showing significant and direct associations of variables. At the same time, all the values in the correlation matrix are below 0.80, ruling out the existence of autocorrelation.

### 5.4. Hypotheses Testing

As far as causality is concerned, the coefficient of determination indicates that around 50% of the variation in the criterion variable is caused by the predicting variables placed in the model of the study (Table 8).

The analysis of the path coefficient reveals that the data supported three of our hypotheses, while two hypotheses were disproved (Table 9). H1, indicating the possibility of improvement in organizational social sustainability (OSS) by the increased level of job safety, is supported. The relationship is significant at *p* = <0.05 as the *t*-statistic (3.955), which is higher than the cutoff value (1.645), indicates the significance of outer model loading and provides sufficient evidence against the null hypothesis. The path coefficient (0.279) shows reasonable strength of the causal relationship between job safety and OSS. Thus, job safety impacts the formation and improvement of organizational social sustainability.

The second hypothesis (H2), assuming the relationship between coworkers’ attitude towards work safety and OSS, is substantiated. The relationship is significant at *p* =< 0.05 and the *T*-test (2.721) rejects the null hypothesis and signifies the outer model loading. The path coefficient (0.181) establishes a positive causal relationship between both variables. The relationship is significant, although not that strong.

The third hypothesis (H3), regarding the supervisor’s role in work safety and improvement in OSS, is supported. The *p*-value of 0.004 establishes the significance of the relationship. The *t*-statistic (2.858) also supports the existence of a relationship and nullifies the null hypothesis. The path coefficient (0.205) confirms the positive impact of the supervisor’s role on the improvement of organizational social sustainability.

The fourth hypothesis (H4) is rejected. Data did not support the conjectured relationship between management work-related safety practices and organizational social sustainability. All the indicators, including *p*-value (0.845), T value (0.195), and path coefficient (0.014), go against the alternative hypothesis; the T value provides strong evidence in favor of the null hypothesis; and the *p*-value nullifies the significance of the proposed relationship.

The fifth hypothesis (H5) is accepted. This hypothesis claimed a relationship between safety programs/policies and organizational social sustainability. The path coefficient (0.148) indicates a positive, although weak, relationship. The *p*-value is just below the cutoff value and, likewise, T statistics are slightly higher than the threshold value, but enough to legalize the hypothesis.

## 6. Discussion

The COVID-19 pandemic has adversely affected almost all aspects of life. We tried our best to keep our study pandemic bias-free; however, certain effects might have penetrated into the study. Hospital administration did not allow us to personally collect data owing to the presence of coronavirus-affected patients in the hospital, and the hospital administration itself arranged data collection for us as per our indicated procedure of data collection. We believe that data was collected properly and no technical weakness existed in data collection. Studies on the impact of the COVID-19 pandemic on healthcare professionals exhibit deep stress, depression, mental health issues, illness, family life issues, and work–family balance-related issues [91,92,93,94,95]. As the respondents were healthcare professionals, they might have been more sensitive regarding work safety, and the history effect might have occurred.

Social sustainability in organizations is a phenomenon broadly recognized as one of the most influential factors in organizations. However, in emerging economies, it is becoming popular in present days [96]. Successful organizations base their competitive advantage on their employees rather than technology and finance [14]. The psychological and cognitive embeddedness of employees in the organization is the formation of social sustainability within the organizations. It is the relatively permanent relationship of employees and the organization that provides an organization with a resource that is neither readily available in the market to buy nor can be emulated easily [81]. Hence this highly subjective, gradual, and pain-staking activity for an organization to develop and improve social sustainability within the organization, as many visible and invisible factors could influence it.

The study produced mixed kinds of expected and unexpected results. Job safety has a profound impact on social sustainability in organizations. It is evident from the results that employees are very concerned about job safety, leaving all other safety concerns behind. Hospital settings are relatively more hazardous and employees are more vulnerable to physical, psychological, chemical, and ergonomic hazards. Hospital waste management in the given context is not that efficient and scientific. Hospital waste management issues can even cause permanent disabilities, particularly for those who work in pathology, radiography, oncology, and surgical departments. Thus, job structure, nature of the task, interaction with the environment, interaction with patients particularly having communicable diseases, and use of chemicals and sharps sometimes become dangerous for the workers. Another job safety-related issue is working in the department of insane, mentally retarded, and mad patients. Health workers working in these departments are prone to even physical violence. In short, a great deal of careful approaches are needed to cope with job safety-related issues, particularly in hospitals. These findings are consistent with the findings of previous studies [19,29,97,98].

The recklessness of employees regarding safety measures is common in the population. Employees seem to be least careful while managing wastes after using chemicals, sharps, rays, disposable equipment, and adopting safety measures in hospitals [99]. In public sector hospitals, management does not provide training on how to work safely inside hazardous environments and what behavior employees should demonstrate to be safe and make others safe [100]. In the same way, hospitals do not offer any awareness programs regarding safety issues and the consequences arising out of them. Employees are not made accountable for any kind of violation of safety standards and the absence of fear of accountability. Our findings, while reflecting the ground realities, explain that coworkers’ carelessness or carefulness directly impact social sustainability in hospitals. The results are consistent with the literature [73,100,101].

In public sector hospitals, workload is relatively high and maintenance of safety standards is not at par [3]. In teaching and tertiary hospitals, dual hierarchies are in action [7]. As such hospitals are affiliated with medical universities, they remain under the influence of the professional hierarchy of the university, apart from the administrative hierarchy of the hospital. In the presence of duality of command, fixing responsibility becomes difficult. This is why supervisory issues usually arise in these hospitals. In the departments of the hospital, the senior registrar (medical doctor) and head nurse are usually assigned supervisory duties. Besides role ambiguity due to the duality of hierarchy, both figures are not trained for understanding and managing work-related safety issues. In these departments, supervisors place more emphasis on curing and treatment of patients, and the caring aspect including safety matters is paid the least attention. Our findings, consistent with those of [102,103,104], suggest that there should be a leading role of a supervisor regarding safety matters that ultimately influence the overall formation and development of social sustainability in an organization.

H4 is rejected, surprisingly. According to our findings, management safety practices have no relationship with social sustainability in healthcare settings. There are no particular management practices in action in departments regarding safety matters. What management wants to happen in the departments is carried out by the supervisors (senior registrar and head nurse). The employees do not have direct interaction with the CEO office or with other senior management regarding safety measures. Thus, supervisors of the department may mediate the relationship between higher management and employees. However, all of them follow the literature, but conversely, some works in the literature mention a significant role of management in putting work safety in place [105,106,107,108].

As far as safety programs and policies are concerned, our data depict that employees attached the least importance to them. This hypothesis is barely accepted. The hospital does not have any formal body to devise, analyze, implement, and evaluate safety-related programs and policies [108]. There are no mechanisms to evaluate the effectiveness of the safety programs and policies in hand. There is a lack of a research-based policy-making process to make the policies more relevant to a given context [109]. Safety policies and programs are usually designed in provincial health departments and circulated to all public sector hospitals for compliance. The unique and individual safety requirements of departments or hospitals are not taken into consideration. However, certain other international/universal safety protocols, for example, safety protocols of the World Health Organization (WHO), are available for use, but, owing to a lack of a proper implantation system, these protocols are seldom seen in action [110]. In short, employees understand the importance of safety-related programs and policies, but, owing to poor implementation structures and processes, they do not completely rely on these programs and policies for their safety.

### Recommendations

After obtaining the statistical results of the study and careful analysis of the responses to the questions, we can present the following recommendations:

In the context of the study, hospitals have many safety issues. These issues arise as a result of a lack of a proper and scientific waste disposal system and implementation of updated safety policies and standards. Thus, hospital administration needs to pay immediate attention to these safety issues.

(a) Employees are very concerned about job safety. Hospital administration must do job safety analysis and job redesign, which are presently lacking, in order to ensure job safety under changing conditions.
(b) Implementation of safety programs and standards is left to ward/department supervisors alone and no involvement of hospital management was noticed as such. Participation of higher management in department-level safety affairs seems necessary to make it more effective and purposeful.
(c) The careless behaviors of employees add to the safety issues in hospitals. Training of employees to make them realize the sensitivity of safety issues and their role in aggravating or mitigating the safety issues is necessary. Apart from training, employees should be held responsible for violations of safety standards.
(d) Supervisors’ training on safety issues is of great importance. Enhancing supervisors’ understanding of safety issues and of different ways to manage them will make a big difference.
(e) Review of safety policies and programs and evaluation of their relevance and effectiveness is of utmost importance. Purging outdated and irrelevant policies, procedures, and standards and introducing appropriate ones is the need of the hour.
(f) A great improvement can be brought in social sustainability in hospitals through managing work safety issues, which will ultimately improve the quality of healthcare services provided to the patients.

## 7. Conclusions

### 7.1. General Conclusion

As the effectiveness of healthcare services is directly contingent upon the competence and attitude of the employees, it is very important to understand and work for the formation and improvement in social sustainability. On the other hand, employees in healthcare industries are more vulnerable to work safety issues as compared with those in other industries. This situation compels employees to think of job switchover. Keeping both of these facts in view, we anchored our study to investigate the role of work safety in improving social sustainability in hospitals. We chose a large public sector hospital for data collection purposes and gathered 431 responses through an adapted questionnaire. Inferential statistics were applied to test the hypotheses. According to our findings (Table 9), employees are very concerned about job safety matters (β = 0.279), followed by the role of a supervisor in ensuring work safety (β = 0.205) and coworkers’ safety-related behaviors (β = 0.181). As senior management has no direct involvement in safety matters (β = 0.014), employees do not give any importance to this aspect. As far as work safety programs and policies are concerned, employees give them the least importance (β = 0.148), because these policies are not renewed, reviewed, and corrected over time to tackle work safety issues. Based on the result of our first hypothesis, we recommend that job analysis and job design be done, keeping in view work safety requirements. As the result of the second hypothesis indicates, frequent training of employees is needed to create awareness of workplace safety issues and reinforce their pro-safety behaviors and minimize their anti-safety behaviors. The result of our third hypothesis shows a key role of supervisors in work safety. As implementation of safety policies, programs, and standards and achieving compliance in response is the sole responsibility of the supervisor, the effectiveness of the supervisor’s role is very important. Thus, training and motivation of supervisors are also necessary as the supervisor’s role is directly related to the management of safety issues. Last, but not the least, the result of our fourth hypothesis indicates that management is not directly involved in safety-related activities in patient departments. Thus, management should participate directly in work safety affairs and programs and policies need to be monitored continuously to ensure their relevancy and effectiveness. All the public sector hospitals in Pakistan are more or less similar as they are run under state institutions. Thus, we can confidently expect that the generalization of the results in hand to other public sector hospitals in the country will be valid.

### 7.2. Implications

Some facts were uncovered in this study. Firstly, the composition of hospital settings in terms of safety provision is unlike others reflected in the literature. Work safety issues are alarming as waste management is not that effective and efficient in these hospitals. Jobs are not safe and employees voiced their concerns regarding job safety. Generally, safety programs and management safety practices are considered to be the guarantors of work safety, but in the given context, both of these mechanisms are dormant. Hospitals neither devise safety policies, programs, and standards according to the contextual requirements, nor does senior management actively participate in safety practices. It is necessary to conduct research to develop indigenous safety policies and programs keeping in view local demands and design such processes that take hospital senior management on board. We suggest managers are involved directly in safety matters and focus on job analysis to make it safe. We further suggest training programs for coworkers and supervisors to enable them to play their due role in ensuring safety at work.

## Figures and Tables

**Table 1:** The items measuring each variable of the study.

Variables	Items (Measurement)	Variables	Items (Measurement)
**Job Safety** Do you agree or disagree that each of the following words or phrases describes your job?	Dangerous	**Management Safety (Practices)** Do you agree or disagree that each of the following words or phrases describes your management?	Provides enough safety training
	Hazardous		Conducts frequent safety inspections
	Unhealthy		Investigates safety problems quickly
	Fear of death		Provides safe equipment
	Chance of death		Provides safe working conditions
	Safe		Helps maintain a clear work area
	Scary		Keeps workers informed of hazards
**Coworker Safety** Do you agree or disagree that each of the following words or phrases describes these people?	Ignore safety rules	**Safety Programs (Policies)** Do you agree or disagree that each of the following words or phrases describes this safety program?	Helps prevent accidents
	Do not care about others’ safety		Unclear
	Pay attention to safety rules		Effective in reducing injuries
	Encourage others to be safe		Does not apply to my workplace
	Keep work area clean		Important
	Safety-oriented		Does not work
**Supervisor Safety** Do you agree or disagree that each of the following words or phrases describes these people?	Praises safe work behaviors	**Organizational Social Sustainability** Do you agree or disagree that each of the following words or phrases describes your relation with the organization?	Sense of belonging
	Keeps workers informed of safety rules		Social capital
			Perceived environment
	Trains workers to be safe		Social interactions/security
	Acts on safety suggestions		Interaction with space
	Updates safety rules		Satisfaction from space
	Enforces safety rules		Voice and influence

**Table 2:** Details of respondents.

Demographics	Frequency	Percentage
Gender		
Male	260	60
Female	171	40
Age (in years)		
18–25	92	21
26–32	102	24
33–40	112	26
41–50	73	17
51 and above	52	12
Experience (in years)		
1–5	90	20
6–10	108	25
11–20	119	28
21–29	81	19
30 and above	33	08
Healthcare Professional		
Doctors	186	43
Nurses	190	44
Others	55	13

**Table 3:** Construct reliability and validity.

Variables *	Cronbach’s Alpha	rho_A	Composite Reliability	(AVE)
Cowrker	0.791	0.833	0.863	0.617
Job	0.870	0.877	0.906	0.659
MgtP	0.739	0.742	0.852	0.658
OSS	0.818	0.819	0.892	0.733
Prgrms	0.779	0.788	0.849	0.531
Supervsr	0.884	0.894	0.915	0.683

* Cowwrker = coworkers, Job = job safety, MgtP = management practices, OSS = organizational social sustainability, Prgrms = safety programs and projects, Supervsr = supervisor, AVE = average variance extracted.

**Table 4:** Discriminant validity (Fornell–Larcker).

	Cowrker	Job	MgtP	OSS	Prgrms	Supervsr
Cowrker	0.786					
Job	0.594	0.812				
MgtP	0.576	0.607	0.811			
OSS	0.579	0.628	0.544	0.856		
Prgrms	0.675	0.758	0.696	0.645	0.759	
Supervsr	0.605	0.588	0.749	0.6	0.722	0.827

**Table 5:** Discriminant validity (Heterotrait–Monotrait (HTMT)).

	Cowrker	Job	MgtP	OSS	Prgrms	Supervsr
Cowrker						
Job	0.698					
MgtP	0.752	0.753				
OSS	0.696	0.734	0.697			
Prgrms	0.848	0.870	0.816	0.779		
Supervsr	0.702	0.667	0.819	0.699	0.814	

**Table 6:** Variance inflation factor (VIF).

	OSS
Cowrker	1.987
Job	2.479
MgtP	2.614
OSS	
Prgrms	3.876
Supervsr	3.036

**Table 7:** Correlations.

	Cowrker	Job	MgtP	OSS	Prgrms	Supervsr
Cowrker	1					
Job	0.694	1				
MgtP	0.676	0.607	1			
OSS	0.679	0.628	0.644	1		
Prgrms	0.675	0.758	0.696	0.645	1	
Supervsr	0.605	0.688	0.749	0.650	0.752	1

**Table 8:** R Square.

	R Square	R Square Adjusted
OSS	0.506	0.498

**Table 9:** Path coefficients.

	Original Sample (O)	Sample Mean (M)	Standard Deviation (STDEV)	T Statistics (|O/STDEV|)	*p* Values
Cowrker -> OSS	0.181	0.184	0.066	2.721	0.007
Job -> OSS	0.279	0.280	0.071	3.955	0.000
MgtP -> OSS	0.014	0.014	0.073	0.195	0.845
Prgrms -> OSS	0.148	0.150	0.082	1.794	0.043
Supervsr -> OSS	0.205	0.203	0.072	2.858	0.004

## Data Availability

The data will be made available on request from the corresponding author.

## References

[1] Stoddard J.E., Pollard C.E., Evans M.R. (2012). The triple bottom line: A framework for sustainable tourism development. Int. J. Hosp. Tour. Adm..

[2] Comber A.J., Brunsdon C., Radburn R. (2011). A spatial analysis of variations in health access: Linking geography, socio-economic status and access perceptions. Int. J. Health Geogr..

[3] Ullah Z., Khan M.Z., Khan M.A. (2020). Towards service quality measurement mechanism of teaching hospitals. Int. J. Healthc. Manag..

[4] Amin M., Nasharuddin S.Z. (2013). Hospital service quality and its effects on patient satisfaction and behavioural intention. Clin. Gov. Int. J..

[5] Swain S., Kar N.C. (2018). Hospital service quality as antecedent of patient satisfaction–a conceptual framework. Int. J. Pharm. Healthc. Mark..

[6] Kruk M.E., Freedman L.P. (2008). Assessing health system performance in developing countries: A review of the literature. Health Policy.

[7] Ullah Z., Khan M.Z., Siddique M. (2017). Analysis of employees’ perception of workplace support and level of motivation in public sector healthcare organization. Bus. Econ. Rev..

[8] Ullah Z., Khan M.Z. (2020). The impact of transactional and transformational leadership on job related outcomes in the nursing profession. Sarhad J. Manag. Sci..

[9] Evans D.B., Edejer T.T., Lauer J., Frenk J., Murray C.J.L. (2001). Measuring quality: From the system to the provider. Int. J. Qual. Health Care.

[10] Ullah Z., Ahmad N. (2020). Study of dynamics of quality of healthcare services: The patient perspective. J. Xi’an Univ. Archit. Technol..

[11] Sugiono N., Ali J., Miranda S. (2020). The effect of employee, management, working environment, and safety culture on occupational healthy and safety performance: A case study in an oil and gas company in Indonesia. Int. J. Integr. Eng..

[12] Morhason-Bello I.O., Odedina F., Rebbeck T.R., Harford J., Dangou J.-M., Denny L., Adewole I.F. (2013). Challenges and opportunities in cancer control in Africa: A perspective from the African Organisation for Research and Training in Cancer. Lancet Oncol..

[13] Rasmussen L.V., Bierbaum R., Oldekop J., Agrawal A. (2017). Bridging the practitioner-researcher divide: Indicators to track environmental, economic, and sociocultural sustainability of agricultural commodity production. Glob. Environ. Chang..

[14] Pfeffer J. (2010). Building sustainable organizations: The human factor. Acad. Manag. Perspect..

[15] Hovlid E., Bukve O., Haug K., Aslaksen A.B., Von Plessen C. (2012). Sustainability of healthcare improvement: What can we learn from learning theory?. BMC Health Serv. Res..

[16] Hussain M., Khan M., Ajmal M., Sheikh K.S., Ahamat A. (2019). A multi-stakeholders view of the barriers of social sustainability in healthcare supply chains. Sustain. Account. Manag. Policy J..

[17] Brugha R., Zwi A. (1998). Improving the quality of private sector delivery of public health services: Challenges and strategies. Health Policy Plan..

[18] Ullah Z., Khan M.Z., Rehman W.U. (2017). Assessing quality of service through customer satisfaction: The case of public sector healthcare organization in pakistan. Ucp Manag. Rev. (UCPMR).

[19] Kortum E., Leka S., Cox T. (2010). Psychosocial risks and work-related stress in developing countries: Health impact, priorities, barriers and solutions. Int. J. Occup. Med. Environ. Health.

[20] Pirzadeh P., Lingard H. (2017). Understanding the dynamics of construction decision making and the impact on work health and safety. J. Manag. Eng..

[21] Hammer L.B., Truxillo D.M., Bodner T., Pytlovany A.C., Richman A. (2019). Exploration of the impact of organisational context on a workplace safety and health intervention. Work Stress.

[22] Che Huei L., Ya-Wen L., Ming Y.C., Chen H.L., Yi W.J., Hung L.M. (2020). Occupational health and safety hazards faced by healthcare professionals in Taiwan: A systematic review of risk factors and control strategies. SAGE Open Med..

[23] Mohd Fahmi M.R. (2017). Using Work Scale Safety (WSS) to Determine Factors Influencing Safety Behavior among Auxiliary Police. Ph.D. Thesis.

[24] Ng L.-P., Choong Y.-O., Kuar L.-S., Tan C.-E., Teoh S.-Y. (2019). Job satisfaction and organizational citizenship behaviour amongst health professionals: The mediating role of work engagement. Int. J. Healthc. Manag..

[25] Azeem M.U., Bajwa S.U., Shahzad K., Aslam H. (2020). Psychological contract violation and turnover intention: The role of job dissatisfaction and work disengagement. Empl. Relat. Int. J..

[26] Seilonen J. (2021). What is Sustainability? A Discourse Analysis of Oil Companies’ Sustainability Reports.

[27] Bibri S.E., Krogstie J. (2017). Smart sustainable cities of the future: An extensive interdisciplinary literature review. Sustain. Cities Soc..

[28] Crespo-Gonzalez C., Benrimoj S.I., Scerri M., Garcia-Cardenas V. (2020). Sustainability of innovations in healthcare: A systematic review and conceptual framework for professional pharmacy services. Res. Soc. Adm. Pharm..

[29] Dzhengiz T. (2020). A literature review of inter-organizational sustainability learning. Sustainability.

[30] Ajmal M.M., Khan M., Hussain M., Helo P. (2018). Conceptualizing and incorporating social sustainability in the business world. Int. J. Sustain. Dev. World Ecol..

[31] Moore J.E., Mascarenhas A., Bain J., Straus S.E. (2017). Developing a comprehensive definition of sustainability. Implement. Sci..

[32] Schaltegger S., Burritt R. (2005). Corporate sustainability. The International Yearbook of Environmental and Resource Economics 2005/2006.

[33] Sodhi M.S., Tang C.S. (2018). Corporate social sustainability in supply chains: A thematic analysis of the literature. Int. J. Prod. Res..

[34] Paulraj A. (2011). Understanding the relationships between internal resources and capabilities, sustainable supply management and organizational sustainability. J. Supply Chain Manag..

[35] Smith P.A., Sharicz C. (2011). The shift needed for sustainability. Learn. Organ..

[36] Milán-García J., Uribe-Toril J., Ruiz-Real J.L., Valenciano J.D.P. (2019). Sustainable local development: An overview of the state of knowledge. Resources.

[37] Smith P.A., Wals A.E., Schwarzin L. (2012). Fostering organizational sustainability through dialogic interaction. Learn. Organ..

[38] Coblentz J.B. (2002). Organizational Sustainability: The Three Aspects that Matter.

[39] Agyeman J., Warner K. (2002). Putting’just sustainability’into place: From paradigm to practice. Policy Manag. Rev..

[40] Fiorino D.J. (2010). Sustainability as a conceptual focus for public administration. Public Adm. Rev..

[41] Hasna A. (2007). Dimensions of Sustainability. J. Eng. Sustain. Dev. Energy Environ. Health.

[42] Opp S.M., Saunders K.L. (2013). Pillar talk: Local sustainability initiatives and policies in the United States—Finding evidence of the “three E’s”: Economic development, environmental protection, and social equity. Urban Aff. Rev..

[43] Stazyk E.C., Moldavanova A., Frederickson H.G. (2016). Sustainability, intergenerational social equity, and the socially responsible organization. Adm. Soc..

[44] Portney K.E., Cuttler Z. (2010). The local nonprofit sector and the pursuit of sustainability in American cities: A preliminary exploration. Local Environ..

[45] Schaltegger S., Burritt R. (2014). Measuring and managing sustainability performance of supply chains. Supply Chain. Manag. Int. J..

[46] Dempsey N., Bramley G., Power S., Brown C. (2011). The social dimension of sustainable development: Defining urban social sustainability. Sustain. Dev..

[47] Koppenjan J.F., Enserink B. (2009). Public–private partnerships in urban infrastructures: Reconciling private sector participation and sustainability. Public Adm. Rev..

[48] Vallance S., Perkins H.C., Dixon J.E. (2011). What is social sustainability? A clarification of concepts. Geoforum.

[49] Lee C.M.J., Che-Ha N., Alwi S.F.S. (2021). Service customer orientation and social sustainability: The case of small medium enterprises. J. Bus. Res..

[50] Sajjad A., Shahbaz W. (2020). Mindfulness and social sustainability: An integrative review. Soc. Indic. Res..

[51] Gálvez A., Tirado F., Martínez M.J. (2020). Work–life balance, organizations and social sustainability: Analyzing female telework in Spain. Sustainability.

[52] Pasaribu S.I., Vanclay F., Zhao Y. (2020). Challenges to implementing socially-sustainable community development in oil palm and forestry operations in Indonesia. Land.

[53] Polèse M., Stren R. (2000). The social sustainability of cities. Chapter.

[54] Nakawuki H. (2019). Job Satisfaction, Occupational Hazards, and Stress and Among Health Workers of Butabika National Referral Mental Hospital. Ph.D. Thesis.

[55] Macik-Frey M., Quick J.C., Nelson D.L. (2007). Advances in occupational health: From a stressful beginning to a positive future. J. Manag..

[56] Wilburn S.Q., Eijkemans G. (2004). Preventing needlestick injuries among healthcare workers: A WHO-ICN collaboration. Int. J. Occup. Environ. Health.

[57] Magnavita N., Chirico F. (2020). New and Emerging Risk Factors in Occupational Health. Appl. Sci..

[58] Wijnen B.F., Lokkerbol J., Boot C., Havermans B.M., Van Der Beek A.J., Smit F. (2020). Implementing interventions to reduce work-related stress among health-care workers: An investment appraisal from the employer’s perspective. Int. Arch. Occup. Environ. Health.

[59] Kwasnicka D., Dombrowski S.U., White M., Sniehotta F.F. (2016). Theoretical explanations for maintenance of behaviour change: A systematic review of behaviour theories. Health Psychol. Rev..

[60] Bogard W.C. (1988). Bringing social theory to hazards research: Conditions and consequences of the mitigation of environmental hazards. Sociol. Perspect..

[61] Reddy V., Bennadi D. (2015). Occupational hazards among dentists: A descriptive study. J. Oral Hyg. Health.

[62] Haleblian J., Finkelstein S. (1999). The influence of organizational acquisition experience on acquisition performance: A behavioral learning perspective. Adm. Sci. Q..

[63] Cox T., Griffiths A., Rial-González E. (2000). Research on Work-Related Stress.

[64] Sauter S.L., Murphy L.R., Hurrell J.J. (1990). Prevention of work-related psychological disorders: A national strategy proposed by the National Institute for Occupational Safety and Health (NIOSH). Am. Psychol..

[65] Whatley M.A., Webster J.M., Smith R.H., Rhodes A. (1999). The effect of a favor on public and private compliance: How internalized is the norm of reciprocity?. Basic Appl. Soc. Psychol..

[66] Widok A.H. (2009). Social sustainability: Theories, concepts, practicability. Environmental Informatics and Industrial Environmental Protection: Concepts, Methods and Tools (2).

[67] Braccini A.M., Margherita E.G. (2019). Exploring organizational sustainability of industry 4.0 under the triple bottom line: The case of a manufacturing company. Sustainability.

[68] Böhringer C., Jochem P.E. (2007). Measuring the immeasurable—A survey of sustainability indices. Ecol. Econ..

[69] Dillard J., Dujon V., King M.C. (2008). Understanding the Social Dimension of Sustainability.

[70] Littig B., Griessler E. (2005). Social sustainability: A catchword between political pragmatism and social theory. Int. J. Sustain. Dev..

[71] Eizenberg E., Jabareen Y. (2017). Social sustainability: A new conceptual framework. Sustainability.

[72] Wilson M.G., DeJoy D.M., Vandenberg R.J., Richardson H.A., McGrath A.L. (2004). Work characteristics and employee health and well-being: Test of a model of healthy work organization. J. Occup. Organ. Psychol..

[73] Nahrgang J.D., Morgeson F.P., Hofmann D.A. (2011). Safety at work: A meta-analytic investigation of the link between job demands, job resources, burnout, engagement, and safety outcomes. J. Appl. Psychol..

[74] Dollard M.F., Bakker A.B. (2010). Psychosocial safety climate as a precursor to conducive work environments, psychological health problems, and employee engagement. J. Occup. Organ. Psychol..

[75] Hayes B.E., Perander J., Smecko T., Trask J. (1998). Measuring perceptions of workplace safety: Development and validation of the work safety scale. J. Saf. Res..

[76] Stromquist N.P. (2002). Education in a Globalized World: The Connectivity of Economic Power, Technology, and Knowledge.

[77] Storper M. (1992). The limits to globalization: Technology districts and international trade. Econ. Geogr..

[78] Jaumotte F., Lall S., Papageorgiou C. (2013). Rising income inequality: Technology, or trade and financial globalization?. IMF Econ. Rev..

[79] Pfeffer J. (1995). Producing sustainable competitive advantage through the effective management of people. Acad. Manag. Perspect..

[80] Bartlett C.A., Ghoshal S. (2002). Building competitive advantage through people. MIT Sloan Manag. Rev..

[81] Boxall P. (1998). Achieving competitive advantage through human resource strategy: Towards a theory of industry dynamics. Hum. Resour. Manag. Rev..

[82] Brown K.A., Willis P.G., Prussia G.E. (2000). Predicting safe employee behavior in the steel industry: Development and test of a sociotechnical model. J. Oper. Manag..

[83] Cella-De-Oliveira F.A. (2013). Indicators of organizational sustainability: A proposition from organizational competences. Int. Rev. Manag. Bus. Res..

[84] Jansen E., Mallan K.M., Daniels L.A. (2015). Extending the validity of the feeding practices and structure questionnaire. Int. J. Behav. Nutr. Phys. Act..

[85] Hoyle R.H., Hoyle R.H. (1995). The structural equation modeling approach: Basic concepts and fundamental issues. Structural Equation Modeling: Concepts, Issues, and Applications.

[86] Henseler J., Ringle C.M., Sarstedt M. (2015). A new criterion for assessing discriminant validity in variance-based structural equation modeling. J. Acad. Mark. Sci..

[87] Wong K.K.-K. (2013). Partial least squares structural equation modeling (PLS-SEM) techniques using SmartPLS. Mark. Bull..

[88] Henseler J. (2017). Bridging design and behavioral research with variance-based structural equation modeling. J. Advert..

[89] Schober P., Boer C., Schwarte L.A. (2018). Correlation coefficients: Appropriate use and interpretation. Anesth. Analg..

[90] Kozak A. (1997). Effects of multicollinearity and autocorrelation on the variable-exponent taper functions. Can. J. For. Res..

[91] Ornell F., Halpern S.C., Kessler F.H.P., Narvaez J.C.D.M. (2020). The impact of the COVID-19 pandemic on the mental health of healthcare professionals. Cad. Saude Publica.

[92] Gavin B., Hayden J., Adamis D., McNicholas F. (2020). Caring for the psychological well-being of healthcare professionals in the Covid-19 pandemic crisis. Ir. Med. J..

[93] Danet A.D. (2021). Psychological impact of COVID-19 pandemic in Western frontline healthcare professionals. A systematic review. Med. Clínica (Engl. Ed.).

[94] Jalili M., Niroomand M., Hadavand F., Zeinali K., Fotouhi A. (2021). Burnout among healthcare professionals during COVID-19 pandemic: A cross-sectional study. Int. Arch. Occup. Environ. Health.

[95] Braquehais M.D., Vargas-Cáceres S., Gómez-Durán E., Nieva G., Valero S., Casas M., Bruguera E. (2020). The impact of the COVID-19 pandemic on the mental health of healthcare professionals. QJM Int. J. Med..

[96] Najjar M., Small M.H., Yasin M.M. (2020). Social sustainability strategy across the supply chain: A conceptual approach from the organisational perspective. Sustainability.

[97] Abid G., Ahmed S., Elahi N.S., Ilyas S. (2020). Antecedents and mechanism of employee well-being for social sustainability: A sequential mediation. Sustain. Prod. Consum..

[98] Epstein M.J., Elkington J., Herman B. (2018). Making Sustainability Work: Best Practices in Managing and Measuring Corporate Social, Environmental and Economic Impacts.

[99] Nwachukwu N.C., Orji F.A., Ugbogu O.C. (2013). Health care waste management–public health benefits, and the need for effective environmental regulatory surveillance in federal Republic of Nigeria. Curr. Top. Public Health.

[100] Gershon R.R., Karkashian C.D., Grosch J.W., Murphy L.R., Escamilla-Cejudo A., Flanagan P.A., Bernacki E., Kasting C., Martin L. (2000). Hospital safety climate and its relationship with safe work practices and workplace exposure incidents. Am. J. Infect. Control.

[101] Clarke S. (2013). Safety leadership: A meta-analytic review of transformational and transactional leadership styles as antecedents of safety behaviours. J. Occup. Organ. Psychol..

[102] Michael J.H., Guo Z.G., Wiedenbeck J.K., Ray C.D. (2006). Production supervisor impacts on subordinates’ safety outcomes: An investigation of leader-member exchange and safety communication. J. Saf. Res..

[103] Harris T.C. (1997). Predicting Workplace Safety Outcomes through Subordinate and Supervisor Involvement in Safety Issues.

[104] Yanar B., Lay M., Smith P.M. (2019). The interplay between supervisor safety support and occupational health and safety vulnerability on work injury. Saf. Health Work.

[105] Thompson R.C., Hilton T.F., Witt L.A. (1998). Where the safety rubber meets the shop floor: A confirmatory model of management influence on workplace safety. J. Saf. Res..

[106] Colligan M.J., Cohen A., Barling J., Frone M.R. (2004). The role of training in promoting workplace safety and health. The Psychology of Workplace Safety.

[107] Ayim Gyekye S. (2005). Workers’ perceptions of workplace safety and job satisfaction. Int. J. Occup. Saf. Ergon..

[108] Khattak F.H. (2009). Hospital waste management in Pakistan. Pak. J. Med. Res..

[109] Abdullah M.T., Shaw J. (2007). A review of the experience of hospital autonomy in Pakistan. Int. J. Health Plan. Manag..

[110] Misbah S., Mahboob U. (2017). Strengths, weaknesses, opportunities, and threats analysis of integrating the World Health Organization patient safety curriculum into undergraduate medical education in Pakistan: A qualitative case study. J. Educ. Eval. Health Prof..

